# Effects of induction on the farrowing process and piglet blood parameters at the time of farrowing^1^

**DOI:** 10.1093/tas/txab032

**Published:** 2021-05-10

**Authors:** Kayla M Mills, Larissa K Shirley, Katharine Sharp, Ricardo Garcia, Aridany Suarez-Trujillo, Kara R Stewart

**Affiliations:** Department of Animal Science, Purdue University, 270 S Russell Street, West Lafayette, IN 47907, USA

**Keywords:** farrowing, induction, piglet

## Abstract

Historically, sows have been induced to farrow using prostaglandin followed by an injection of oxytocin 24 h later. Benefits of induction can include decreased rate of stillbirths, dystocia, and postnatal mortality along with increasing the likelihood of farrowings being attended. Several studies have indicated that oxytocin administration may negatively impact fetal oxygen supply during parturition, potentially from umbilical cords breaking prior to birth, resulting in increased preweaning mortality. Therefore, the objective of this study was to determine if various induction protocols impact umbilical cord breakage and fetal blood parameters at birth. Fifty-eight primiparous and multiparous sows were assigned to one of three treatments: no induction (NO; *n* = 24) or 2 cc prostaglandin administered on day 114 of gestation followed by either 1 cc of oxytocin 24 h later (OXY24; *n* = 13) or 0.5 cc of oxytocin at 6 and 12 h after prostaglandin (OXY6; *n* = 21). Details of the farrowing process were recorded, and umbilical cord blood was collected from piglets at birth and evaluated on an iSTAT machine using an Abbott EC8+ test cartridge. There were no differences in total born, number born alive, stillborns, mummies, or assistance needed during farrowing. Induced sows were more likely to farrow by day 115 compared to naturally farrowing sows (*P* = 0.02). Sows in the OXY24 treatment tended to have longer farrowings when compared to both NO and OXY6 (4.8 vs. 3.6 vs. 3.9 h; *P* = 0.09). Colostrum from OXY6 sows tended to have a greater amount of lactose present than NO and OXY24 (*P* = 0.05). Colostrum from sows with longer gestation lengths had a higher percentage of fat (*P* = 0.03). Piglets born from NO sows had higher base excess, total carbon dioxide, and glucose, which suggests that these piglets had prolonged moments of asphyxiation (*P* < 0.01). OXY24 piglets had the lowest blood pH which is indicative of hypoxic birthing conditions (*P* < 0.01). Preweaning mortality was driven largely by a low birth weight coupled with low colostrum intake (*P* = 0.03). All piglets, regardless of treatment, displayed signs of stress during farrowing. Induction did not influence preweaning mortality but has the potential to decrease the incidence by increasing attended farrowings.

Genetic selection for highly prolific sows and increased total born has consequently resulted in longer farrowing duration and higher occurrences of low birth weight piglets, which negatively impacts preweaning survival through decreased colostrum intake (Le Dividich, 1999; Devillers et al., 2011; Kirkden et al., 2013; Gourley et al., 2020). Longer farrowing duration increases the risk of hypoxia in piglets born later in the litter because of a risk of umbilical occlusion or breaking of the cord (Randall, 1972a, 1972b; English and Wilkinson, 1982; Alonso-Spilsbury et al., 2005). Hypoxic events during farrowing can lead to low viability of the piglet, which impairs suckling ability and decreases the chance of surviving until weaning (English and Wilkinson, 1982; Herpin et al., 1996; Kammersgaard et al., 2011; Gourley et al., 2020).

One common farrowing induction method is to administer prostaglandin followed by an injection of oxytocin 24 h later. Benefits of induction with prostaglandin and oxytocin include decreased rate of stillbirths, dystocia, decreased farrowing duration, and postnatal mortality along with the possibility of increasing the chance of farrowings being attended (Straw et al., 2008; Nguyen et al., 2011). A greater number of sows farrowed during the work day when given oxytocin 6 or 24 h after prostaglandin when compared to sows who only received prostaglandin, which improved the predictability, at the start of farrowing (Alexopoulos et al., 1998; Kirkwood and Aherne, 1998). It has also been shown that it is not necessary to wait 24 h to administer oxytocin but rather to give it in multiple, low dosages to achieve a decrease in stillbirths and postnatal mortality (Clark and Bilkei, 2002). While several studies have indicated that oxytocin administration during the farrowing process may negatively impact fetal oxygen supply at parturition (Mota-Rojas et al., 2002; Ward et al., 2019), none have evaluated inducing farrowing on fetal oxygenation. Therefore, the objective of this study was to compare the impacts of various induction protocols on umbilical cord breakage, fetal blood parameters at birth, colostrum intake, and preweaning mortality.

## MATERIALS AND METHODS

### Animals

#### Sows.

All procedures involving animals were reviewed and approved by the Institutional Animal Care and Use Committee (PACUC #1902001856). Confirmed pregnant sows were moved into farrowing crates on day 112 of gestation at the Animal Sciences Research and Education swine facility at Purdue University. On day 114 of gestation, 58 sows were blocked by parity (gilt-parity 1, and parity 2+) and assigned one of three treatments: no induction (NO; *n* = 24), 2 cc prostaglandin (Lutalyse, Zoetis Inc., Parsippany-Troy Hills, NJ) administered on day 114 of gestation followed by 1 cc of oxytocin 24 h later (OXY24; *n* = 13), or 2 cc prostaglandin administered on day 114 of gestation followed by 0.5 cc of oxytocin at 6 and 12 h after prostaglandin (OXY6; *n* = 21). Administration of prostaglandin was performed at 1000 h intramuscularly in the neck of each sow using an 18-gauge, 40-mm needle. Oxytocin was administered subcutaneously in the crease between the vulva and ham using a 20-gauge, 12-mm needle. Data collected included induction-to-farrowing interval, farrowing duration, farrowing assistance, time of birth for each piglet, umbilical cord breakage, number of piglets born alive, stillborns, mummies, and whether feed was present in the feeder at farrowing. Umbilical cords were classified as broken if the piglet’s cord was no longer attached to the mother at birth.

#### Colostrum composition.

Within 12 h of the birth of the first piglet, a 50-mL colostrum sample was collected by milking the front, middle, and back teats and stored in −80 ºC until further analysis for total fat, protein, and lactose. Prior to analysis, all samples were thawed at 4 °C overnight in a continuous rotator in order to homogenize samples. Fat percentage was measured in triplicate by loading samples in a nonheparinized capillary tube, which were centrifuged at 12,000 × *g* for 10 min. Length of the fat layer on the top and the total sample length were measured using a caliper (Pittsburgh Automotive, Camarillo, CA). Fat percentage was calculated as the ratio between fat length and total sample length, multiplied by 100. Protein concentration was determined by diluting homogenized colostrum with phosphate buffered saline (1:100). Ten microliters of colostrum were mixed with 250 uL of Bradford reagent (Pierce Coomassie Plus Assay Kit, Thermo Fisher Scientific, Waltham, MA). Color development in samples and standard curve (bovine serum albumin, 0.025–2 mg/mL range) was measured with a spectrophotometer (Sparks 10-M multimode microplate reader, Tecan, Männedorf, Switzerland). Lactose was extracted from 200 µL of skimmed colostrum by adding 800 µL acetonitrile. Extracts were centrifuged at 3,220 × *g* at room temperature for 10 min to remove precipitated proteins. Before injecting samples, they were diluted 1:5 with acetonitrile and spiked with 1,000 ng of D-lactose-13C (Sigma-Aldrich, St. Louis, MO). Liquid chromatography was performed using Intrada Amino Acid 3 μm, 2 × 150 mm column (Imtrakt USA, Portland, OR) connected to an Agilent 6470 QQQ LC-MS/MS system (Agilent, Santa Clara, CA). Acetonitrile with 0.3% of formic acid and acetonitrile with 100 mM ammonium formate solution (20:80 v/v) were used as mobile phases.

#### Sow and piglet blood parameters.

Blood samples were collected from the ear veins of sows at the time of the birth of the first and sixth piglets and the end of farrowing and evaluated for glucose (Glu) and hemoglobin (Hgb) levels using an AimStrip *Plus* blood glucose monitor (Germaine Laboratories, Inc., San Antonio, TX) and the HemoCue Hb 201+ Hemoglobin Analyzer (HemoCue America, Brea, CA). The end of farrowing was characterized by placental membranes expelled from the sow. Sows were provided manual assistance if the birthing interval between piglets exceeded 30 min. All farrowings were attended 24 h a day until all sows farrowed.

At the time of birth of each piglet (NO, *n* = 118; OXY, *n* = 105; OXY6, *n* = 79), the umbilical cord blood was collected and immediately placed in a Microtainer Lithium Heparin/PST Gel 600-uL tube (Becton, Dickinson and Company, Franklin Lakes, NJ) and inverted to prevent clotting. Blood samples were analyzed with the iSTAT portable clinical analyzer using the EC8+ cartridge (Abbott, Priceton, NJ) for sodium (Na), potassium (K), ionized calcium (iCa), Glu, Hematocrit (Hct), Hgb, pH, partial pressure of carbon dioxide (pCO_2_), partial pressure of oxygen (pO_2_), base excess (BE), bicarbonate (HCO3), total carbon dioxide (tCO_2_), and oxygen saturation (sO_2_). Piglets were dried using bath towels and allowed to nurse normally. Piglets were weighed at birth and 24 h after to calculate colostrum intake using the equation previously described (Devillers et al., 2011). The equation uses the change in body weight between birth and 24 h coupled with the number of potential minutes a piglet has to suckle colostrum as a quantitative measure of colostrum intake. Crossfostering within treatment was performed after 24 h to standardize lactation litter sizes. Piglets were weighed again at weaning to calculate the average daily gain (ADG) from birth to weaning, and preweaning mortality was recorded for each litter.

### Statistical Analysis

Analysis of farrowing variables from the sow was performed using the MIXED procedure of SAS 9.4 (SAS Inst. Inc., Cary, NC) with treatment and parity as main effects. Binomial variables were analyzed using the GLIMMIX procedure. Sow blood Glu and Hgb were evaluated using the MIXED procedure for repeated measures with treatment and parity as fixed effects and whether the sow ate prior to farrowing as a covariate in the model. The MIXED procedure of SAS 9.4 was also used to evaluate piglet blood parameters using treatment, parity, and birth order as main effects and sow as a random effect. Birth order classes were created to evaluate birth order as a main effect: birth order class 1 (piglets 1–5); birth order class 2 (piglets 6–10); birth order class 3 (piglets born after the 10th piglet). Parity classes were also created as follows: parity class 1 (gilts and parity 1) and parity class 2 (parity 2+). For colostrum quality analysis, the MIXED procedure of SAS was used with treatment as a fixed effect and gestation length as a covariate. A Tukey’s means comparison was used to determine differences among means for piglet data and colostrum quality. Significance was defined as *P* < 0.05 and values of 0.05 ≤ *P* ≤ 0.10 were considered tendencies.

## RESULTS

### Effect of Induction on the Farrowing Process and Sow Blood Parameters

There were no differences among treatments in the time from induction to farrowing, total born, number born alive, stillborns, mummies, or assistance needed during farrowing (Table 1). OXY24 animals tended to have longer farrowings (*P* = 0.096). Interestingly, sows that were parity 2+ required more assistance than parities 0–1 (23.8% vs. 6.5 %, respectively, *P* < 0.001). Although treatment did not have an effect on the percentage of sows who farrowed during the work day or within 36 h of induction, sows were more likely to farrow by day 115 who were induced compared to naturally farrowing sows (Table 1).

**Table 1.** Effect of induction protocol on day 114 of gestation on the farrowing process

Treatment	NO^*a*^	OXY24^*b*^	OXY6^*c*^	SE	*P*-value
*n*	24	13	21		
Farrowing length, hrs	3.55	4.76	3.86	1.13	0.096
Farrowing interval^*d*^, hrs	n/a	38.20	41.01	6.22	0.973
Number born alive	11.90	12.39	11.43	0.77	0.626
Stillborn, animals	0.71	0.69	0.76	0.32	0.845
Mummies, animals	0.54	0.69	0.71	0.27	0.820
Pulled pigs, piglets	1.833	1.461	1.29	0.71	0.918
Assistance, %	13.33	13.80	14.94	3.84	0.787
Cord broken, %	24.00	29.20	20.48	4.66	0.389
% Farrowed by day 115	30.43^b^	78.57^a^	66.67^a^	11.38	0.021
% Farrowed by 36 h	n/a	78.57	66.67	11.38	0.573
% Farrowed during work day (0600 to 1600 h)	34.78	14.29	38.10	10.86	0.898

^*a*^No induction.

^*b*^2cc Lutalyse given on day 114 of gestation followed by 1 cc oxytocin 24 h later.

^*c*^2cc Lutalyse given on day 114 of gestation followed by 0.5 cc oxytocin 6 and 12 h later.

^*d*^Farrowing *interval is defined as the time between Lutalyse injection and the start of farrowing.*

^*a,b*^Means within a column with different superscripts differ (P ≤ 0.05).

Sow blood Hgb remained constant throughout farrowing regardless of treatment (Table 2). Younger parities had higher levels of Hgb on average than older parities (Table 2). Blood Glu levels were not affected by treatment; however, older parity sows had a greater increase in Glu levels at the end of farrowing than younger sows (*P* = 0.035).

**Table 2.** The effect of induction protocol and parity on sow blood Glu and Hgb during the farrowing process

Treatment	NO^*a*^		OXY24^*b*^		OXY6^*c*^			*P*-value				
Parity class	1^*d*^	2^*e*^	1	2	1	2	SE	Trt	Time	Parity class	Trt × Parity class	Time × Parity class
Sows that ate prior farrowing, %^*f*^	27	50	12	100	38	57	11	–	–	–	–	–
Glu pig 1, mg/dL	81.82	79.58	74.00	85.67	69.69	80.14	5.816	0.963	<0.001	0.035	0.152	0.994
Glu pig 6, mg/dL	80.89	79.80	70.63	83.75	74.00	80.29	3.775					
Glu end, mg/dL	83.70	85.00	84.89	87.00	83.67	96.50	8.631					
Hgb pig 1, g/L	112.0	103.3	118.1	96.75	115.4	106.5	5.375	0.925	0.1988	0.005	0.636	0.228
Hgb pig 6, g/L	108.0	100.4	111.8	99.25	105.2	106.0	4.521					
Hgb end, g/L	115.9	100.4	109.4	102.3	112.2	106.0	5.672					

^*a*^No induction.

^*b*^2cc Lutalyse given on day 114 of gestation followed by 1 cc oxytocin 24 h later.

^*c*^2cc Lutalyse given on day 114 of gestation followed by 0.5 cc oxytocin 6 and 12 h later.

^*d*^Parity class 1: parities 0 and 1.

^*e*^Parity class 2: parity 2+.

^*f*^Feeders were evaluated at the start of farrowing to determine if the sow had eaten or not.

#### Induction and preweaning mortality.

Induction did not influence preweaning mortality, colostrum intake, or ADG from birth to weaning (Table 3). On average, piglets born from OXY24 sows had a lighter birth weight on average (Table 3). Preweaning mortality was largely driven by birth weight and, consequently, colostrum intake (*P* < 0.001) with survivability increasing with higher birth weights and colostrum intake (Table 4). Animals born between 0 and 1 kg consumed 214.7 g of colostrum on average, which is just above the recommended minimum of 200 g (Devillers et al., 2011). These animals also had the highest rate of preweaning mortality with 49% of these animals dying before weaning (Table 4). Birth order did not influence preweaning mortality in the present study. Although not significant, it is interesting to note that piglets born from sows who farrowed over 4 h had a numerical increase in preweaning mortality (22% vs. 15%; *P* = 0.363) and consumed about 15 g less of colostrum on average (*P* = 0.208). Average daily gain from birth to weaning was influenced by colostrum intake and birth weight where lighter pigs consumed less colostrum and, therefore, gained less (*P* < 0.001). It was also observed that animals who died prior to weaning lost weight on average compared to piglets that survived (0.235 vs. −0.014; *P* < 0.001). Induction did not influence fat or protein content in colostrum, but OXY6 tended to have a greater amount of lactose present when compared to NO and OXY24 (Table 5). There was also a tendency for an interaction between treatment and gestation length for the percentage of lactose (Table 5). Gestation length influenced the percentage of fat with the higher fat content being associated with a longer gestation length (Table 5).

**Table 3.** Effect of induction on day 114 of gestation, birth weight, farrowing length, and colostrum intake on preweaning mortality

					*P*-value					
Treatment	NO^*a*^	OXY24^*b*^	OXY6^*c*^	SE	Trt	Birth weight class	Trt × Birth weight class	Farrowing length class	Colostrum intake	Colostrum intake × Birth weight class
Preweaning mortality, %	17.25	19.19	17.84	3.011	0.189	<0.001	0.423	0.256	0.001	<0.001
Colostrum intake, g	316.8	301.4	334.3	5.428	0.505	<0.001	0.582	0.004	–	–
Birth weight, kg	1.345^a^	1.299^b^	1.428^a^	0.026	<0.001	–	–	–	–	–
ADG^*d*^, kg/d	0.200	0.186	0.204	0.009	0.600	0.055	0.825	0.017	0.005	0.294

^*a*^No induction.

^*b*^2cc Lutalyse given on day 114 of gestation followed by 1 cc oxytocin 24 h later.

^*c*^2cc Lutalyse given on day 114 of gestation followed by 0.5 cc oxytocin 6 and 12 h later.

^*d*^ADG from birth to weaning.

^*a,b*^Means within a column with different superscripts differ (*P* ≤ 0.05).

**Table 4.** Effect of birth weight on preweaning mortality

					*P*-value			
Birth weight class	0–1 kg (*n* = 109)	1–1.5 kg (*n* = 318)	>1.5 kg (*n* = 241)	SE	Trt	Birth weight class	Colostrum intake	Birth weight class × Colostrum intake
Preweaning mortality, %	49.54	11.64	13.28	4.811	0.940	<0.001	<0.001	0.030
Colostrum intake, g	214.7^c^	300.0^b^	383.6^a^	3.999	–	–	–	–

^*a–c*^Means within a column with different superscripts differ (*P* ≤ 0.05).

**Table 5.** Effect of induction on day 114 of gestation on measures of colostrum quality

	NO^*a*^	OXY24^*b*^	OXY6^*c*^	SE	Trt	Gestation length	Trt × Gestation length
Gestation length, days	116.0	115.5	115.8	0.424	0.595	–	–
Lactose, %	1.394	1.851	2.170	0.311	0.053	0.112	0.058
Fat, %	11.00	10.05	9.424	0.887	0.150	0.032	0.156
Protein, %	9.554	8.868	8.997	0.468	0.386	0.904	0.381

^*a*^No induction.

^*b*^2cc Lutalyse given on day 114 of gestation followed by 1 cc oxytocin 24 h later.

^*c*^2cc Lutalyse given on day 114 of gestation followed by 0.5 cc oxytocin 6 and 12 h later.

#### Induction and its effects on piglet blood parameters at birth.

Piglets born from sows who were in the OXY6 treatment group had the same blood K level as OXY24, the same blood Glu as NO, intermediate BE, and intermediate pH. Piglets from OXY24 mothers had the lowest pH, BE, and Glu coupled with the highest amount of pO_2_ (Table 6). Piglets from older parity mothers showed greater amounts of Glu in the blood as well as BE, tCO_2_, and lower sO_2_.

**Table 6.** Effect of induction on day 114 of gestation, parity, and birth order on piglet blood parameters at birth

												*P*-value		
Treatment	NO^*a*^	OXY24^*b*^	OXY6^*c*^	SE	PC1^*d*^	PC2^*e*^	SE	OC1^*f*^	OC2^*g*^	OC3^*h*^	SE	Trt	Parity	Birth order
*n*	118	105	79		174	128		123	109	70				
Na, mmol/L	128.4	128.1	129.5	0.775	127.6	129.9	0.615	128.3	129.0	128.6	0.834	0.609	0.021	0.625
K, mmol/L	6.738^b^	7.600^a^	7.672^a^	0.214	7.455	7.035	0.173	7.130	7.315	7.473	0.242	<0.001	0.023	0.480
iCa, mmol/L	1.771	1.652	1.684	0.044	1.749	1.650	0.032	1.661	1.728	1.756	0.045	0.058	0.090	0.406
Glu, mg/dL	35.93^a^	26.04^b^	32.68^a^	1.687	29.31	34.78	1.295	28.93^b^	32.53^ab^	35.00^a^	1.797	<0.001	<0.001	0.006
Hct, % PCV	23.52	24.22	25.53	0.966	23.52	25.37	0.661	24.37	23.88	24.77	0.901	0.526	0.040	0.107
Hgb, g/dL	8.548	8.803	9.299	0.316	8.723	8.971	0.213	8.747	8.656	9.291	0.265	0.271	0.537	0.081
pH	7.354^a^	7.297^b^	7.339^ab^	0.017	7.316	7.349	0.014	7.340	7.318	7.331	0.017	<0.001	0.161	0.758
pCO_2_, mmHg	53.84^b^	58.17^a^	55.17^ab^	2.151	55.06	56.57	1.566	56.44	56.48	53.14	1.985	0.007	0.302	0.446
pO_2_, mmHg	121.8^ab^	126.1^a^	101.3^b^	6.412	125.3	107.9	5.081	119.9	114.0	120.6	6.282	0.023	0.107	0.773
BE, mmol/L	3.802^a^	1.760^b^	2.462^ab^	0.592	1.349	4.635	0.540	3.744^a^	2.729^ab^	1.014^b^	0.758	0.016	<0.001	0.009
HCO_3_, mmol/L	29.10	28.10	27.99	0.539	27.46	29.83	0.527	29.16^a^	28.69^ab^	26.91^b^	0.649	0.435	<0.001	0.021
tCO_2_, mmol/L	31.00	29.87	29.54	0.518	29.10	31.75	0.518	31.09^a^	30.36^ab^	28.50^b^	0.679	0.264	<0.001	0.006
sO_2_, %	91.43	89.56	85.22	2.244	90.75	86.95	1.763	88.23	88.35	91.94	1.794	0.198	0.211	0.556

^*a*^No induction.

^*b*^2cc Lutalyse given on day 114 of gestation followed by 1 cc oxytocin 24 h later.

^*c*^2cc Lutalyse given on day 114 of gestation followed by 0.5 cc oxytocin 6 and 12 h later.

^*d*^Parities 0 and 1.

eParity 2+.

^*f*^First to fifth piglet born.

^*g*^Sixth to tenth piglet born.

^*h*^Born after 10 piglets.

^*a,b*^Means within a column with different superscripts differ (*P* ≤ 0.05).

Piglets born latest in the litter had the highest blood Glu levels (Table 6). Interestingly, piglets born earlier in the litter had a higher BE and tCO_2_ than those born later (*P* = 0.009). Piglets born from greater parity sows who farrowed naturally had higher BE, tCO_2_, pCO_2_, and Glu (Table 7). Piglets born to older sows in the OXY6 treatment group had an increased Hgb and Hct.

**Table 7.** Effect of induction and birth parity on piglet blood parameters at birth

Treatment	NO^*a*^		OXY24^*b*^		OXY6^*c*^			*P*-value
Parity class	1^*d*^	2^*e*^	1	2	1	2	SE	Trt × Parity
*n*	74	44	68	37	32	47		
Na, mmol/L	126.9	131.0	128.6	127.2	127.2	131.0	1.448	0.064
K, mmol/L	6.958	6.368	7.678	7.451	8.161	7.349	0.324	0.530
iCa, mmol/L	1.850^a^	1.639^b^	1.650^b^	1.656^b^	1.723^ab^	1.657^ab^	0.070	0.047
Glu, mg/dL	35.26^a^	37.07^a^	24.36^b^	29.09^b^	26.09^b^	37.17^a^	2.420	0.003
Hct, % PCV	23.92^b^	22.84^b^	24.37^b^	23.91^ab^	20.71^b^	28.78^a^	1.294	0.002
Hgb, g/dL	8.779^ab^	8.171^b^	8.909^ab^	8.603^ab^	8.086^ab^	9.891^a^	0.486	0.034
pH	7.325^abc^	7.402^ac^	7.309^bc^	7.273^b^	7.309^abc^	7.360^abc^	0.033	0.003
pCO2, mmHg	53.25^b^	54.82^b^	55.61^b^	62.87^a^	58.14^ab^	53.17^b^	4.119	0.002
pO_2_, mmHg	131.8	104.9	125.1	128.1	111.0	94.77	10.03	0.271
BE, mmol/L	1.466^b^	7.767^a^	0.853^b^	3.472^ab^	2.161^b^	2.660^b^	1.109	0.014
HCO_3_, mmol/L	27.51^b^	31.80^a^	27.11^b^	29.98^a^	28.11^ab^	27.91^b^	1.055	0.009
tCO_2_, mmol/L	29.14^b^	34.16^a^	28.78^b^	31.92^a^	29.74^ab^	29.40^b^	0.964	0.001
sO_2_, %	93.79	87.31	88.98	90.61	87.32	83.83	3.807	0.512

^*a*^No induction.

^*b*^2cc Lutalyse given on day 114 of gestation followed by 1 cc oxytocin 24 h later.

^*c*^2cc Lutalyse given on d114 of gestation followed by 0.5 cc oxytocin 6 and 12 h later.

^*d*^Parities 0 and 1.

^*e*^Parity 2+.

^*a–c*^Means within a column with different superscripts differ (*P* ≤ 0.05).

## DISCUSSION

Inducing farrowing on day 114 of gestation had no negative effects on the farrowing process or litter characteristics, which is consistent with previous research using different induction protocols (Otto et al., 2017; Vallet and Miles, 2017). It is important to note that all farrowings in this study were attended where sows were checked every 30 min and assistance in farrowing provided, resulting in a low incidence of stillbirths. Being able to schedule farrowings may help producers in making staffing decisions for heavier farrowing days, thus creating a greater likelihood of farrowings being attended. In this study, we observed that induced sows were more likely to farrow by day 115 but not during the working day hours, which contradicts previous findings. This contradicts previous research evaluating the use of prostaglandin combined with the use of oxytocin which showed that sows were more likely to farrow during the work day (Alexopoulos et al., 1998; Kirkwood and Aherne, 1998); however, both trials used cloprostenol as their prostaglandin source, whereas we used dinoprost. Although induced sows farrowed on average 38–41 h after the initial prostaglandin injection, there was a lot of variation in the time that farrowing initiated. More research is needed to increase the synchrony of the initiation of farrowing following induction in order to increase the likelihood of attendance during farrowing without 24-h staffing.

Partial pressure of carbon dioxide, pH, HCO_3_, and BE are all related to each other and signify events of asphyxia (Englehart and Schreiber, 2006). Base excess serves as a marker for lactic acid, which is an indicator of increased incidents of asphyxia (Englehart and Schreiber, 2006). Blood Glu can also serve as a marker of the neonate undergoing asphyxia (Herpin et al., 1996). Piglets who have higher levels of lactate in their blood at birth are more likely to die prior to weaning, which is consistent with hypoxic events decreasing viability and negatively impacting suckling after birth (English and Wilkinson, 1982; Gourley et al., 2020). Therefore, if an animal has high blood levels of BE, pCO_2_, and Glu coupled with low HCO_3_ and pH, this is an indicator that the animal is deprived of oxygen during the birthing process.

Piglets born earlier in the litter had lower levels of blood Glu, which suggest that they had a better oxygen supply and is consistent with previous work (Gourley et al., 2020). Interestingly, piglets born earlier in the litter had a higher BE and tCO_2_ than those born later and is contradictory from previous research (Herpin et al., 1996). Piglets born from sows who farrowed naturally and of older parities had higher BE, tCO_2_, pCO_2_, and Glu, which suggests that these piglets had prolonged moments of asphyxiation.

Packed cell volume (Hct) and Hgb are correlated (Turkson and Ganyo, 2015) and positively associated with hypoxic events in the body (Zhao et al., 2018). We observed an increase in Hct and Hgb in piglets who were born from older parity sows, sows in the OXY6 treatment, and piglets that were born later in the litter, which may have experienced a decreased oxygen supply at birth.

Sodium, potassium, and calcium are all integral in the pathway associated with hypoxic events (Gusarova et al., 2011). In order to maintain ATP levels in the cell, Na and K transport is inhibited across the cell membrane (Gusarova et al., 2011). Consequently, hypoxia induces the release of calcium to inhibit the entire process (Gusarova et al., 2011). In our piglets, we noted that calcium tended to be highest and K was lowest in piglets born from naturally farrowing gilts.

Despite these few alterations in piglet blood indicators of hypoxic events, piglet ADG and survivability to weaning was not affected by farrowing induction. Similar to many other research trials, preweaning survival was driven by birth weight and colostrum intake (Le Dividich, 1999; Devillers et al., 2011; Gourley et al., 2020).

Although treatment did not influence preweaning mortality or colostrum intake, it is interesting to note that the percentage of lactose was higher in colostrum from OXY6 sows, which indicates that the secretory activation in the mammary gland was happening sooner than the other treatments. Similar increase of lactose in colostrum was found in sows induced with alphaprostol (Foisnet et al., 2011). In that specific case, the greater concentration of lactose in colostrum of induced sows was related to greater prolactin in blood. Further studies should be performed to measure the effect of these induction protocols on maternal hormones. Colostral fat percentage was affected by the gestation length and not by treatment. Shorter gestation lengths have been shown to be related to lower concentrations of colostral fat (Jackson et al., 1995). Colostrum composition mean values were slightly different compared to previous authors (Klobasa et al., 1987; Zhang et al., 2018), possible due to the different methods used to determine lactose, protein, and fat percentages. The techniques used in the present study were previously validated and used by Collares et al. (1997) and Chen et al. (2017). Whether oxytocin administration decreases available colostrum to the neonate, especially those born later in the litter, is not known and warrants future research since this could impact growth and reproductive performance for these animals later in life. Sows injected with carbetocin, a long-lasting analogue of oxytocin, after the birth of the first piglet showed a reduced output of colostrum, but this timing of oxytocin is different than in the present study and the mechanism behind this remains unknown (Ward et al., 2019).

## Conclusion

In conclusion, induction does not negatively impact the farrowing process or fetal blood oxygen at birth. Birth weight and colostrum intake were the main drivers of preweaning mortality in this study with ADG from birth to weaning being higher in animals who survived. Although birth order did not impact preweaning mortality or colostrum intake, piglets born later in the litter did show signs of asphyxia, which may impact overall lifetime growth and development of the animal. Both induction protocols and natural farrowings showed at least some signs of asphyxia, but it did not increase preweaning mortality or ADG after birth. Therefore, induction can be used to encourage sows to farrow by day 115 of gestation without negatively impacting piglets.
